# Astrocyte *Fabp7* modulates nocturnal seizure threshold and activity-dependent gene expression in mouse brain

**DOI:** 10.1093/pnasnexus/pgaf146

**Published:** 2025-05-07

**Authors:** Micah Lefton, Carlos C Flores, Yuji Owada, Christopher J Davis, Thomas N Ferraro, Yool Lee, Wheaton L Schroeder, Jason R Gerstner

**Affiliations:** Elson S. Floyd College of Medicine, Washington State University, Spokane, WA 99202, USA; Elson S. Floyd College of Medicine, Washington State University, Spokane, WA 99202, USA; Department of Organ Anatomy, Graduate School of Medicine, Tohoku University, Seiryo-cho 2-1, Aobaku, Sendai 980-8575, Japan; Elson S. Floyd College of Medicine, Washington State University, Spokane, WA 99202, USA; Sleep and Performance Research Center, Washington State University, Spokane, WA 99202, USA; Steve Gleason Institute for Neuroscience, Washington State University, Spokane, WA 99202, USA; Department of Biomedical Sciences, Cooper Medical School of Rowan University, Camden, NJ 08103, USA; Elson S. Floyd College of Medicine, Washington State University, Spokane, WA 99202, USA; Sleep and Performance Research Center, Washington State University, Spokane, WA 99202, USA; Steve Gleason Institute for Neuroscience, Washington State University, Spokane, WA 99202, USA; Voiland College of Engineering and Architecture, Washington State University, 305 NE Spokane St, Pullman, WA 99164, USA; Elson S. Floyd College of Medicine, Washington State University, Spokane, WA 99202, USA; Sleep and Performance Research Center, Washington State University, Spokane, WA 99202, USA; Steve Gleason Institute for Neuroscience, Washington State University, Spokane, WA 99202, USA

**Keywords:** excitability, transcription, circadian, blbp, glia

## Abstract

Epileptic seizures often track with time of day and/or changes in vigilance state; however, specific molecular and cellular mechanisms driving the ictal and temporal associations are lacking. Astrocytes are a type of glial cell known to modulate neuronal excitability and circadian rhythms. These cells also abundantly express fatty acid–binding protein 7 (Fabp7), a clock-driven molecule necessary for normal sleep regulation, lipid signaling, and gene transcription. To determine whether Fabp7 influences time-of-day-dependent seizure susceptibility, we tested male C57/BL6N wild-type (WT) and *Fabp7* knockout (KO) mice using electroshock seizure threshold. Compared with WT mice, *Fabp7* KO mice exhibited markedly higher general- and maximal-electroshock seizure thresholds (GESTs and MESTs, respectively) during the dark phase, but not the light phase. We used RNA-seq to determine the role of Fabp7 in activity-dependent gene expression in nocturnal seizures and compared genome-wide mRNA expression in cortical/hippocampal tissue collected from WT-MEST and *Fabp7* KO-MEST mice with WT-SHAM and *Fabp7* KO-SHAM mice during the dark period. Whereas significant differential expression of immediate early genes was observed in WT-MEST compared with WT-SHAM, this effect was blocked in the *Fabp7* KO-MEST versus *Fabp7* KO-SHAM. Gene ontology and pathway analysis of all groups revealed significant overlap between WT-MEST:WT-SHAM and *Fabp7* KO-SHAM:WT-SHAM comparisons, suggesting basal mRNA levels of core molecular and cellular mechanisms in the brain of *Fabp7* KO approximate postictal WT brain. Together, these data suggest that Fabp7 regulates time-of-day-dependent neural excitability and that neural activity likely interacts with astrocyte Fabp7-mediated signaling cascades to influence activity-dependent gene expression.

## Introduction

Epilepsy is a neurological disorder affecting around 65 million people worldwide, and studies over the past two decades have revealed that glial cells may play a crucial role in seizure etiology with strong therapeutic potential for treatment (1, 2). About 90% of drug-resistant epilepsy patients exhibit a circadian regulation of seizures, independent of the epilepsy type or brain region, which may also be influenced by sleep/wake behavior (3). Astrocytes, a type of glial cell, are known to affect neural excitability, changes in sleep/wake state, and circadian rhythms (4); however, how these systems functionally interact to influence seizure susceptibility remains largely unknown. Recently, pathways regulating lipid-accumulated reactive astrocytes were shown to promote disease progression in epilepsy (5). Our previous studies have revealed that the astrocyte-enriched lipid-binding protein, fatty acid–binding protein 7 (Fabp7), is regulated by core circadian clock components (6, 7) and regulates sleep across phylogenetically disparate species, from flies to mice to humans (8). Given *Fabp7* gene expression was elevated in dendritic layers of the hippocampus by kainate-induced seizures (9), Fabp7 may represent a molecular node for integrating changes in sleep/wake state, circadian rhythms, and lipid metabolism in astrocytes and neural progenitors with changes in seizure propensity (10, 11). Here, we characterize time-of-day differences in electroshock seizure threshold in wild-type (WT) and *Fabp7* knockout (KO) mice, changes in cortical/hippocampal gene expression, and subsequent gene ontology (GO) and pathway analyses in cross comparisons between WT-SHAM, WT-MEST, *Fabp7* KO-SHAM, and *Fabp7* KO-MEST mice.

## Results

**Fig. 1.**
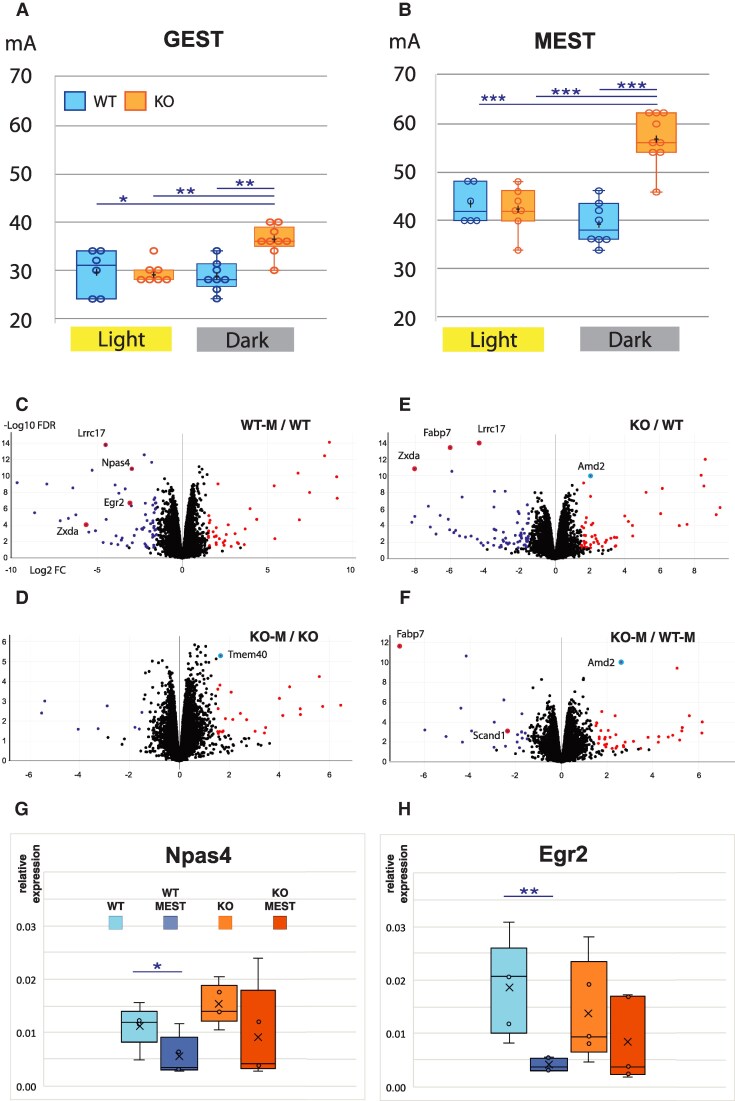
*Fabp7* KO mice have increased nocturnal seizure threshold associated with differential activity–dependent gene expression compared with WT mice. A and B) General and maximal seizure thresholds for WT and KO mice during light and dark periods are plotted. Data for the two light time points and the two dark time points are combined: WT light (*n* = 6), *Fabp7* KO light (*n* = 7), WT dark (*n* = 8), and *Fabp7* KO dark (*n* = 9). One-way ANOVA, *P* = 0.0008 (GEST) and *P* < 0.0001 (MEST); post hoc Bonferroni, *P < 0.05, **P < 0.01, ***P < 0.001. C–F) Volcano plots of DEGs from RNA-seq results of cortical/hippocampal tissue for ZT20 MEST and SHAM mice. Log2 of fold change is plotted on the *x*-axis and –Log10 FDR is plotted on the *y*-axis. G and H) Plots of qPCR results (relative expression = 2^−ΔCt^), with *β-*actin as a normalization control for WT, WT-MEST, KO, and KO-MEST using the same five biological replicates as for RNA-seq. WT versus WT-MEST comparisons showed a significant decrease with MEST (unpaired two-tailed t test, *Naps4 P* = 0.045; *Egr2 P* = 0.006) but no significance for KO versus KO-MEST (unpaired, two-tailed t test, *Naps4 P* = 0.202; *Egr2 P* = 0.354).

We first determined time-of-day dynamics in seizure threshold in WT and *Fabp7* KO mice. Our experimental design included measuring seizure threshold at two time points in the light phase (ZT4 and ZT8) and the dark phase (ZT18 and ZT20). We observed a significant difference in seizure threshold in both generalized (GEST) and maximal (MEST) seizures (*P* = 0.0008 and *P* < 0.0001, respectively, one-way ANOVA), with a time-of-day-dependent change in GEST and MEST in *Fabp7* KO compared with WT (Fig. 1A and B).

As elevated seizure threshold levels in *Fabp7* KO versus WT mice were specific to the dark phase, we characterized alterations of bulk cortical/hippocampal tissue transcriptomic signatures using RNA-seq during the nocturnal period for each genotype following recurring electroshock seizures and comparing them to control mice (SHAM) without seizures. Analysis of mRNA expression between WT-MEST and WT-SHAM identified many differentially expressed genes (DEGs; *n* = 3,857; false discovery rate [FDR] < 0.05), including several immediate early genes (IEGs), such as *Npas4* and *Egr2* (Fig. 1G and H, SI Appendix, Dataset S1), recapitulating previous findings (12). However, when we evaluated *Fabp7* KO-MEST compared with *Fabp7* KO-SHAM, we did not observe nearly as many DEGs (*n* = 201; FDR < 0.05), and many DEGs/IEGs identified in WT-MEST versus WT-SHAM were not affected by seizures in the *Fabp7* KO-MEST compared with *Fabp7* KO-SHAM mice (Fig. 1D, SI Appendix, Dataset S1). We then compared *Fabp7* KO-SHAM to WT-SHAM, which uncovered 2,577 DEGs (FDR < 0.05), including the genetic background control *Fabp7* mRNA (Fig. 1E). Unexpectedly, levels of DEGs in WT-MEST versus WT-SHAM (Fig. 1C, SI Appendix, Dataset S1) were similarly affected in *Fabp7* KO-SHAM to WT-SHAM, including *Lrrc17* and *Zxda* (Fig. 1E, SI Appendix, Dataset S1). Lastly, we compared *Fabp7* KO-MEST with WT-MEST, which identified 1,570 DEGs (FDR < 0.05), of which only a few overlapped in *Fabp7* KO-SHAM versus WT-SHAM (e.g. *Fabp7* and *Amd2*; Fig. 1F, SI Appendix, Dataset S1).

**Fig. 2.**
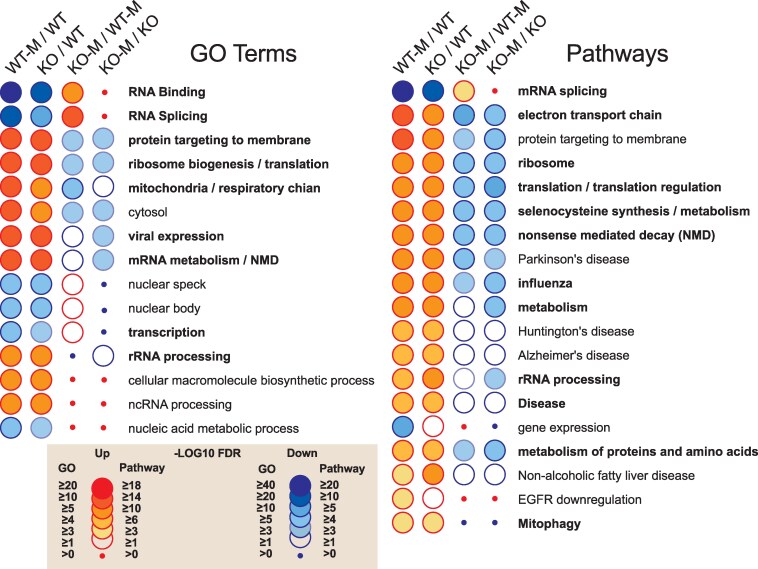
GO and pathway analyses of DEGs in various comparisons between WT-MEST, WT-SHAM, KO-MEST, and KO-SHAM. GO and pathway overrepresentation reveal similar patterns between WT-MEST/WTSHAM and KO-SHAM/WT-SHAM. Many of these GO and pathway patterns were reversed in the KO-MEST/WTMEST and KO-MEST/KO-SHAM comparisons. Threshold of the GO terms and pathways is FDR < 0.1.

GO and pathway analysis revealed that RNA splicing and RNA binding were among the top overrepresented from down-regulated genes between the WT-MEST versus WT-SHAM as well as the *Fabp7* KO-SHAM versus WT-SHAM comparisons, suggesting common transcriptional programming among these groups (Fig. 2, SI Appendix, Dataset S2). Overrepresentation among these two comparisons in up-regulated GO and pathways included protein targeting to membrane, ribosomal regulation, translation, and mitochondrial respiration and electron transport (Fig. 2). This similarity included neurodegenerative disease pathways, such as Parkinson's, Huntington's, and Alzheimer's diseases. Remarkably, almost all of these shared overrepresented GO and pathways between WT-MEST:WT-SHAM and *Fabp7* KO-SHAM:WT-SHAM were inversely overrepresented and shared between *Fabp7* KO-MEST:WT-MEST and *Fabp7* KO-MEST:*Fabp7*-SHAM groups (Fig. 2).

## Discussion

We observed a significantly higher GEST and MEST in the nocturnal (wake) period of *Fabp7* KO compared with WT mice (Fig. 1A and B), which corresponded with disruption of seizure-associated DEGs (Fig. 1C–F, SI Appendix, Dataset S1) and their pathways (Fig. 2, SI Appendix, Dataset S2). The lack of significant diurnal effects on WT-MEST could be due to differences in genetic background and/or sampling times on different light–dark schedules compared with our previous study (13). Recently, local wake slow-wave (LoWS) activity showed progressive adaptive responses following network excitability prior to interictal epileptiform discharges (IEDs), which reduced the impact of subsequent IEDs (14). How these LoWS changes relate to variability in network seizure paths on circadian and slower timescales in patients with focal epilepsy remains to be determined (15). Comparing wake-dependent network activity relationships in electroencephalograph signatures between *Fabp7* KO and WT mice before and after seizures merits future investigation and may support an epilepsy homeostasis hypothesis, wherein LoWS reduces aberrant brain activity (14). In summary, these results show that *Fabp7* is required for normal neural activity–dependent molecular and cellular processes and suggest that nocturnal astrocyte-regulated *Fabp7*-mediated signaling cascades are necessary for seizure responses. Given that about 30% of epilepsy patients eventually progress to a drug-resistant state, with glial scar formation and reactive glia at the epileptic focus involving astrocyte-derived lipid transport mechanisms (5), the discovery of a role for Fabp7 in regulating seizures and seizure-associated pathways may represent a novel therapeutic target to treat certain intractable forms of epilepsy. Future studies using pharmacological seizure models may help determine the underlying Fabp7-dependent mechanisms responsible for changes in seizure susceptibility.

## Materials and methods

*Fabp7* KO and WT mice were housed on a 12:12 h light:dark cycle with access to food and water ad libitum. Mice were subjected to GEST and MEST (Fig. 1A and B) or SHAM, with cortical/hippocampal tissue harvested and used for RNA-seq (Fig. 1C–F) and GO and pathway analysis (Fig. 2). All studies were approved by the Institutional Animal Care and Use Committees at the Washington State University (WSU; ASAF #6509; see SI Appendix, Extended Materials and Methods).

## Supplementary Material

pgaf146_Supplementary_Data

## Data Availability

RNA-seq data are deposited in NCBI GEO (accession no. GSE271985).
