# Supplementary material for: Astrocyte Fabp7 modulates nocturnal seizure threshold and activity-dependent gene expression in mouse brain
Source: PNAS Nexus. 2025 May 7;4(5):pgaf146. doi: 10.1093/pnasnexus/pgaf146 (PMC12082287; doi:10.1093/pnasnexus/pgaf146)
Supplement: pgaf146_Supplementary_Data [file pgaf146_supplementary_data.zip › PNASNEXUS-PNASNEXUS-2024-01387R-s03.pdf]

PNAS nexus

Supplemental Materials and Methods for

Astrocyte *Fabp7* modulates nocturnal seizure threshold and activity-dependent gene expression in mouse brain

Micah Lefton[1t], Carlos C. Flores[1t], Yuji Owada[2], Christopher J. Davis[1,3,4], Thomas N. Ferraro[5], Yool Lee[1,3,4], Wheaton L. Schroeder[6], and Jason R. Gerstner[1,3,4*]

[1]Elson S. Floyd College of Medicine, Washington State University, Spokane, WA 99202, USA
[2]Department of Organ Anatomy, Graduate School of Medicine, Tohoku University, Seiryo-cho 2-1, Aobaku, Sendai 980-8575, Japan
[3]Sleep and Performance Research Center, Washington State University, Spokane, WA 99202, USA
[4]Steve Gleason Institute for Neuroscience, Washington State University, Spokane, WA 99202, USA
[5]Department of Biomedical Sciences, Cooper Medical School of Rowan University, Camden, New Jersey, USA
[6]The Gene and Linda Voiland School of Chemical Engineering and Bioengineering, Washington State University, Pullman, WA 99164, USA

[t]Co-first author
*Corresponding author: Jason R. Gerstner

Email: j.gerstner@wsu.edu

Author Contributions: Conceptualization: J.R.G.; Data curation: M.L.; C.C.F.; Formal analysis: M.L., C.C.F., J.R.G.; Funding acquisition: C.J.D., J.R.G. Investigation: M.L., C.C.F.; Methodology: C.C.F.; T.N.F.; J.R.G.; Project administration: C.C.F., J.R.G.; Resources: Y.O.; C.J.D.; J.R.G.; Supervision: C.C.F.; J.R.G.; Writing—original draft: J.R.G.; Writing—review and editing: M.L., C.C.F., Y.O., C.J.D., T.N.F., Y.L., W.L.S., J.R.G.; All authors reviewed and approved the final version of the manuscript.

Competing Interest Statement: J.R.G. is founder of Blood Brain Biotechnology, LLC.

Classification: Biological, Health, and Medical Sciences/Neuroscience

Keywords: excitability, transcription, circadian, blbp, glia.

This PDF file includes:

Extended Materials and Methods Text

Extended Materials and Methods

Animals

All studies were approved by the Institutional Animal Care and Use Committees at the Washington State University (WSU; ASAF #6509) in accordance with the guidelines of the US National Institutes of Health. Experiments involved C57BL/6N wild type mice (The Jackson Laboratory, Bar Harbor, ME) and coisogenic *Fabp7* knockout (KO) mice (from Y. Owada) and were bred in-house at the WSU Health Sciences Campus vivarium. *Fabp7* KO mice were backcrossed to the C57BL/6N genetic background for at least 6 generations and maintained as a homozygous strain. Litters were weaned between 21–22 days and pups were group housed by sex until the age of 13-16 weeks when they were entered into the study. Mice were maintained on a 12:12 hour light:dark cycle (lights on Zeitgeber time (ZT) 0, lights off ZT12) with access to food and water *ad-libitum*.

Seizure tests and tissue dissection

Due to the variable effect of estrous cycle on seizure susceptibility (3), only male mice were studied. WT and *Fabp7* KO mice (N=6-9 per group and condition) were tested for seizure threshold at 4 timepoints (ZT4, ZT8, ZT18, and ZT20), using a single electric shock delivered via ear clip electrodes once per day. SHAM mice received the same handling but did not receive the shock. We used a constant current electroshock unit (model No. 7801, Ugo Basile, Varese, Italy) in which the initial current level was set at 20 mA and increased by 2 mA with each successive daily trial until a maximal seizure, defined by bilateral tonic hind limb extension was observed. Other parameters of the stimulus were held constant (60 Hz, 0.4 ms pulse width, 0.2 s duration). Upon MEST, mice were euthanized, and brains were harvested immediately from WT-MEST (N=5), *Fabp7* KO-MEST (N=5), WT-SHAM (N=5), and *Fabp7* KO-SHAM mice (N=4) at ZT20 and flash frozen, and kept at -80C until processing. Unilateral brain tissue encompassing the hippocampus and cortex (-1 to 3 mm AP and 0 to 2.5 mm ML) was blocked on dry ice, and homogenates used for subsequent RNA extraction, library construction, Illumina sequencing and data analysis.

RNA isolation, cDNA synthesis, and high throughput sequencing

Total RNA was purified using the RNeasy Mini Kit (Qiagen). The integrity of total RNA was assessed using Fragment Analyzer (Advanced Analytical Technologies, Ankeny, IA) with the High Sensitivity RNA Analysis Kit. RNA samples with RQNs ranging from 8 to 10 were used for RNA library preparation with the TruSeq Stranded mRNA Library Prep Kit (Illumina, San Diego, CA). Briefly, mRNA was isolated from 1-2.5 µg of total RNA using poly-T oligo attached to magnetic beads and then subjected to fragmentation, followed by cDNA synthesis, dAtailing, adaptor ligation and PCR enrichment. The sizes of RNA libraries were assessed by Fragment Analyzer with the High Sensitivity NGS Fragment Analysis Kit. The concentrations of RNA libraries were measured using the StepOnePlus Real-Time PCR System (ThermoFisher Scientific, San Jose, CA) with the KAPA Library Quantification Kit (Kapabiosystems, Wilmington, MA). DNA was sequenced from both ends (paired-end) with a read length of 150 bp. The raw bcl files were converted to fastq files using the software program bcl2fastq, and adaptors were trimmed from the fastq files during the conversion. Sequence data (FASTQ files) were processed by trimming low-quality reads using Trimmomatic (version 0.39). and removing rRNA sequences using SortMeRNA (version 4.3.5). The remaining reads were aligned to the Mus musculus reference genome (mm10, UCSC) using HISAT2 (version 2.2.1). Gene expression quantification was analyzed using featureCounts (part of the Subread package, version 2.0.3).

qPCR

Quantitative PCR was performed on cDNA prepared for RNA-seq, with 5 biological replicates per genotype/condition. For each 20µl reaction, five ng of cDNA was added to SYBR-Green Master Mix (APExBIO, Houston, TX) and primers were added to a final concentration of 0.5 µM. A *β-actin* primer pair was used as a control for normalization. Reactions were run on an MJ Research PTC-200 Thermocycler with the following parameters: 95°C for 10 minutes, then 41 cycles of 95°C for 15s, 55°C for 10s and 60°C for 30s. Primers used were: *Npas4* forward TCTTGCCTGCATCTACACTCGC; Npas4 reverse TCCAGGTAGTGCTGCCACAATG; *Egr2* forward CCTTTGACCAGATGAACGGAGTG; *Egr2* reverse CTGGTTTCTAGGTGCAGAGATGG; *β-actin* Forward CATCCGTAAAGACCTCTATGCCAAC; *β-actin* reverse ATGGAGCCACCGATCCACA.

Data analysis

Generalized and maximal seizures were expressed as arithmetic mean values for each experimental group. One-Way ANOVAs were used to examine the effect of time of day on seizure thresholds. Post hoc analyses to examine statistical relationships for seizure threshold values between individual groups were conducted using the Bonferroni test (Prism stats package, v5). For analysis of seizure data, comparisons were collapsed to lights-on and lights-off phases since there were no significant differences between groups within each phase. Differential expression, volcano plots, GO and Pathway analysis of our data were conducted using Biojupies (https://maayanlab.cloud/biojupies/). qPCR analysis was performed using relative expression ($2^{-\Delta Ct}$) values with β-*actin* as a normalization control. The significance of differences was assessed by unpaired, two-tailed t-tests (Prism stats package, v5).
